# Photoconductivities in MoS_2_ Nanoflake Photoconductors

**DOI:** 10.1186/s11671-016-1331-y

**Published:** 2016-03-02

**Authors:** Wei-Chu Shen, Ruei-San Chen, Ying-Sheng Huang

**Affiliations:** Department of Electronic Engineering, National Taiwan University of Science and Technology, Taipei, 10607 Taiwan; Graduate Institute of Applied Science and Technology, National Taiwan University of Science and Technology, Taipei, 10607 Taiwan

**Keywords:** Molybdenum disulfide, Layer semiconductor, Nanostructure, Photoconductivity, Responsivity, Gain

## Abstract

Photoconductivities in molybdenum disulfide (MoS_2_) layered nanostructures with two-hexagonal crystalline structure prepared by mechanical exfoliation were investigated. The photoconductor-type MoS_2_ nanoflakes exhibit remarkable photoresponse under the above bandgap excitation wavelength of 532 nm at different optical intensity. The photocurrent responsivity and photoconductive gain of nanoflakes can reach, respectively, 30 AW^−1^ and 103 at the intensity of 50 Wm^−2^, which are several orders of magnitude higher than those of their bulk counterparts. The vacuum-enhanced photocurrent and power-independent responsivity/gain indicate a surface-controlled photoconduction mechanism in the MoS_2_ nanomaterial.

## Background

Molybdenum disulfide (MoS_2_), a layer semiconductor with an indirect bandgap of 1.2 eV, has been studied most frequently among the big material family of transition metal dichalcogenide (TMD) [1]. Distinct from their bulk counterparts, recently, MoS_2_ monolayers exhibiting direct-bandgap property and enhanced photoluminescence efficiency due to the quantum confinement effect have been discovered [2, 3]. This finding opens up a brand new research direction for TMD layer semiconductors as the building blocks for optoelectronic applications. Integration of MoS_2_ and graphene could also realize the full two-dimensional (2D) material heterostructures [4], which is an ideal system for the development of next-generation ultrathin, flexible, and transparent light-emitting [5], light-harvesting [6, 7], and light-detecting devices [8, 9].

Taking the advantages of 2D structure and high quantum efficiency [7], the MoS_2_ monolayers and multilayers prepared by mechanical exfoliation and coating techniques have been demonstrated to be an active material for light-detecting devices [10–16]. Among them, most reports investigated the photodetectors based on the configuration of field-effect transistor (FET). The 2D MoS_2_ phototransistors exhibit ultrahigh responsivity and rapid response to the light in the visible range. However, fundamental photoconduction (PC) properties in the pristine MoS_2_ and its nanostructures were rarely investigated. Here, we report on a comparative study of the photoconductor-type MoS_2_ nanoflakes and their bulk counterparts. The photoconduction performance was investigated by quantitatively defining responsivity and photoconductive gain. The mechanism was also discussed by the light intensity- and environment-dependent PC measurements.

## Methods

The MoS_2_ layer crystals used for this study were grown using the chemical vapor transport (CVT) method using bromine (Br) as the transport agent [17]. The source material powders including molybdenum and sulfur together with the bromine were sealed in a quartz ampoule at a vacuum degree of 2 × 10^−5^ Torr. Prior to the CVT growth process, the ampoules were annealed at 1050 °C in an oven for 1 month to compound the source materials. The temperatures of the source and crystallization ends were, respectively, controlled at 1050 and 960 °C during the CVT growth. The MoS_2_ nanoflakes were obtained by exfoliating bulk crystals using dicing tape and were then dispersed on the insulating SiO_2_ (300 nm)/*n*-Si templates with pre-patterned Ti/Au circuits. Two platinum (Pt) metal contacts were subsequently deposited on the selected MoS_2_ flakes using focused-ion beam (FIB) technique. The voltage and current of the ion beam for the Pt precursor decomposition were operated at 30 kV and 100 pA, respectively. In addition, silver paste was used for the metal electrode of the millimeter-sized MoS_2_ bulks. The crystal quality of MoS_2_ was characterized using field-emission scanning electron microscopy (FESEM), Raman spectroscopy, and X-ray diffractometry (XRD). The thicknesses of the MoS_2_ flakes were defined by the atomic force microscopy (AFM). Electrical characterization was performed at an ultralow current leakage probe station (LakeShore Cryotronics TTP4). The dc voltage and current were, respectively, sourced and measured by a semiconductor characterization system (Keithley 4200-SCS). A Nd:YAG laser with a wavelength of 532 nm was used as an excitation light source for the photoconductivity measurement. An optical diffuser was used to broaden laser beam size (~20 mm^2^) to uniformly illuminate the conduction channel of the nanoflake and bulk samples for the steady-state photocurrent measurements. The incident laser power was measured by a calibrated power meter (Ophir Nova II) with a silicon photodiode head (Ophir PD300-UV).

## Results and Discussion

**Fig. 1:**
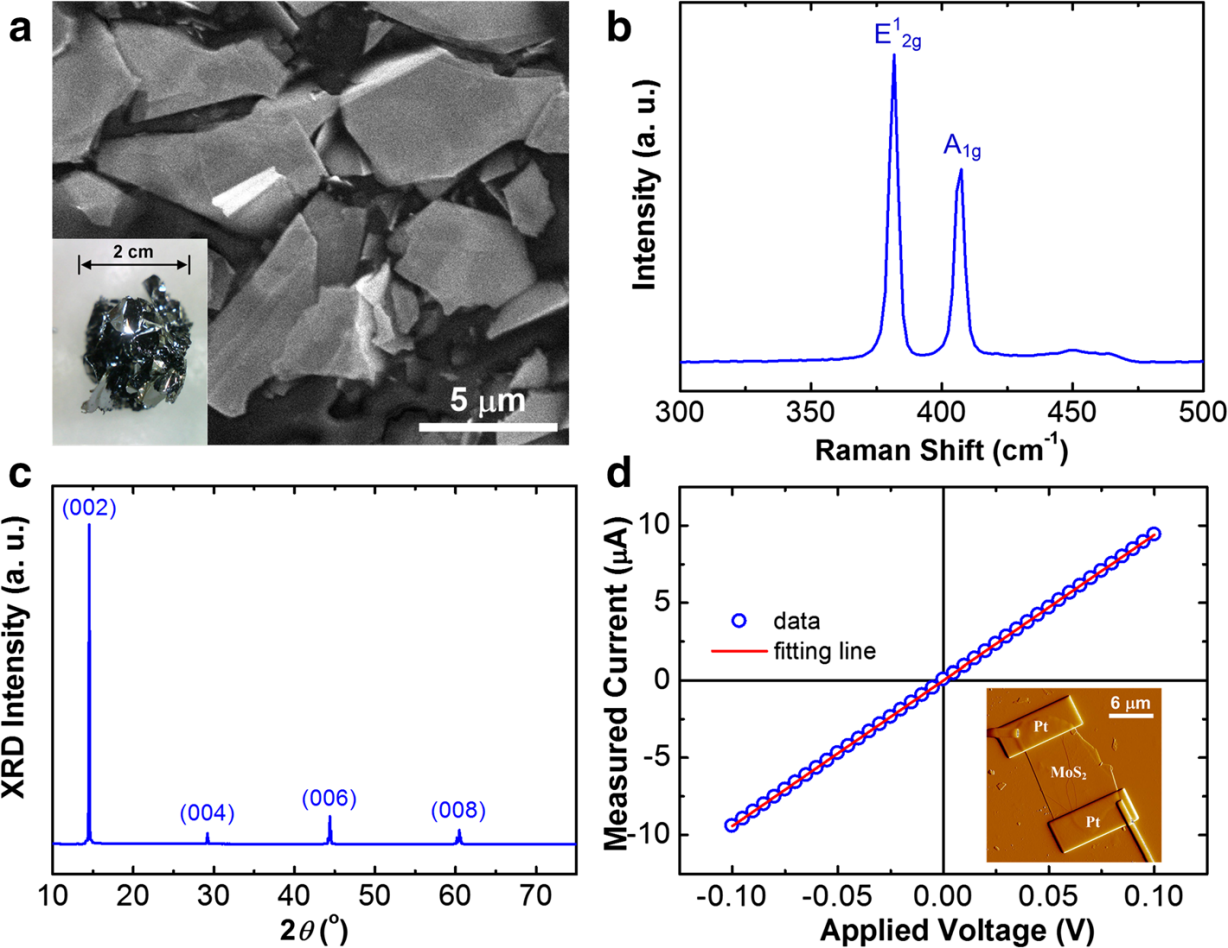
**a** FESEM image of MoS_2_ flakes on the dicing tape after preliminary mechanical exfoliation. *Inset*: photo of a MoS_2_ bulk crystal cluster taken directly from the quartz ampoule after CVT growth. **b** Raman spectrum and **c** XRD pattern of MoS_2_ bulk crystal. **d** *I*-*V* curve for the two-terminal MoS_2_ nanoflake with a thickness of 66 ± 6 nm. *Inset*: the corresponding AFM image of the MoS_2_ nanoflake device fabricated using the FIB approach

Figure 1a depicts a FESEM image of the MoS_2_ flakes after preliminary mechanical exfoliation. The flakes on the dicing tape show irregular shapes, and their area sizes were reduced to micrometer scale from the millimeter-sized bulk crystals. A photo in the inset of Fig. 1a shows a cluster of MoS_2_ single crystals taken directly from the quartz ampoule after CVT growth. Figure 1b depicts a Raman spectrum of a MoS_2_ single crystal using the light source of 414.5 nm wavelength. Two major peaks were observed, and their peak positions determined by curve fitting are at 382 and 407 cm^−1^, which are, respectively, consistent with the E^1^_2g_ and A_1g_ modes for the 2H-MoS_2_ [18]. The full width at half maximum (FWHM) values of the Raman peaks are 2.7 (E^1^_2g_) and 3.7 (A_1g_) cm^−1^. The XRD measurement was also used to characterize the structural quality of the MoS_2_ layer crystals. Figure 1c depicts an XRD pattern with four diffraction peaks centered at 14.5°, 29.2°, 44.4°, and 60.4°. These peaks are indexed as (002), (004), (006), and (008) diffraction planes, respectively, according to the database (JCPDS #872416). The single one out-of-plane orientation of 〈001〉 (*c*-axis) further confirms the crystalline quality of the 2H-MoS_2_.

In addition, the electric contacts of MoS_2_ nanoflake devices were examined by the two-terminal current versus voltage (*I*-*V*) measurement. Figure 1d depicts a representative *I*-*V* curve for the MoS_2_ nanoflake with a thickness of 66 ± 6 nm. The linear *I*-*V* relationship indicates a good ohmic contact condition of the photoconductor-type device. The details of ohmic contact fabrication using FIB technique for the TMD layered nanostructures can be found in our earlier publications [19, 20]. The corresponding AFM image of the nanoflake device is also shown in the insets of Fig. 1d.

**Fig. 2:**
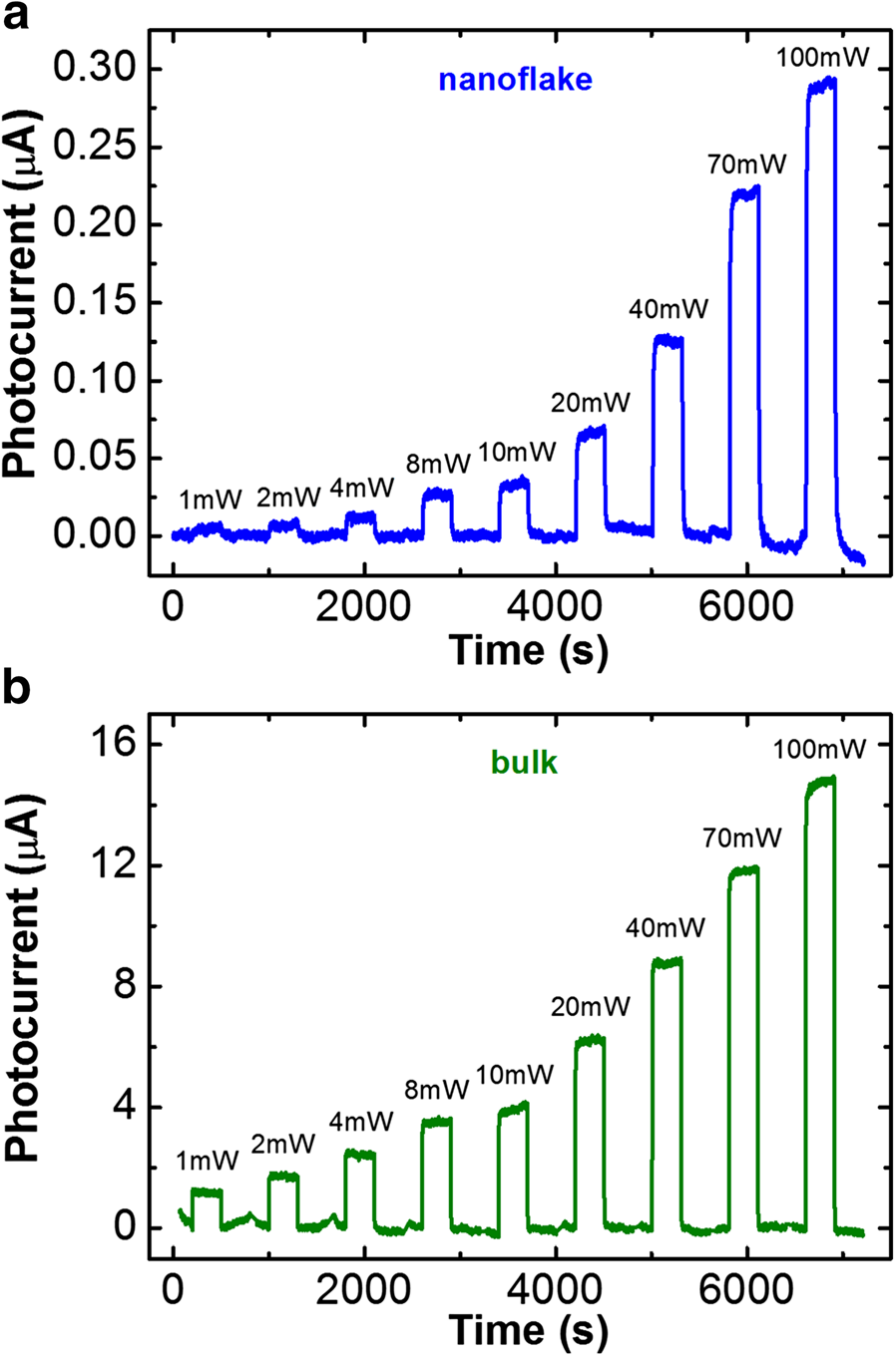
**a** Photocurrent responses to the different light power and the excitation wavelengths of 532 nm measured in air ambience for **a** the MoS_2_ nanoflake (*t* = 45 nm) and **b** the bulk crystal (*t* = 63 μm)

Photocurrent responses under the excitation of 532 nm wavelength (*λ*) at a bias of 0.1 V and at different laser powers for a MoS_2_ nanoflake (*t* = 45 nm) were shown in Fig. 2a. For comparison, the photoresponse measurement under the same excitation condition at a bias of 1 V for a bulk crystal (*t* = 63 μm) was also performed and is shown in Fig. 2b. All the background dark currents were subtracted from the response curves to reveal the photocurrent values. The results show that both the nanoflake and the bulk exhibit clear photoresponse to the different excitation power and the photocurrent increases with an increase of power.

**Fig. 3:**
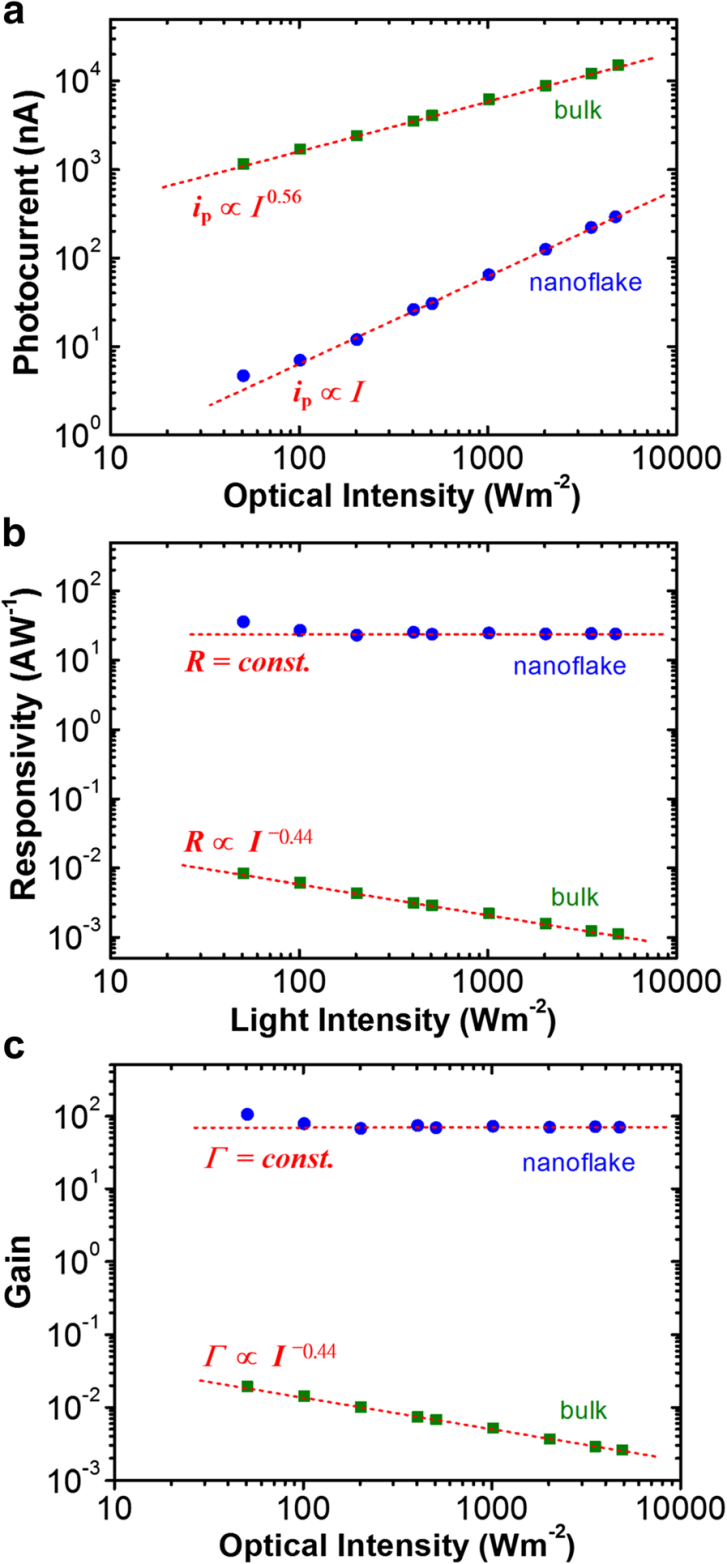
**a** Photocurrent, **b** responsivity, and **c** photoconductive gain as a function of light intensity at 532 nm excitations for the MoS_2_ nanoflake (*t* = 45 nm) and the bulk crystal (*t* = 63 μm)

The difference in photocurrent level between the nanoflake and bulk can be observed by the plot of photocurrent (*i*_p_) versus intensity (*I*), Fig. 3a. From the result, it is noticed that the overall photocurrent of the bulk is approximately one to two orders of magnitude higher than that of the nanostructure. In addition, the photocurrent value is linearly dependent on the intensity, i.e., *i*_p_ ∝ *I*, for the nanoflake. The intensity-dependent behavior is different from the bulk. The photocurrent is less sensitive to the increase in light intensity, and their relationship follows a power law of *i*_p_ ∝ *I*^*β*^, where *β* = 0.56, for the bulk.

Photoconduction is a two-step process that consists of light absorption and carrier collection. Photocurrent value depends on the extrinsic properties of device such as applied bias and electrode inter-distance and the intrinsic properties of material such as carrier mobility and lifetime. To understand the performance of the MoS_2_ photoconductors and their underneath mechanism, two critical parameters including photocurrent responsivity and photoconductive gain were investigated.

The responsivity (*R*) value is a measure of photocurrent generation efficiency of a photodetector and is defined as the photocurrent generated by the power of light incident on an effective area of a photoconductor (*P*). Therefore, responsivity is written as

1 $$ R = \frac{i_p}{P}, $$

where *P* = *IA* = *Iwl*, where *A* is the projected area of the photoconductor (*A* = *wl*), *w* is the width, and *l* is the length of the photoconductor [21]. Figure 3b depicts the calculated responsivity as a function of light intensity. The result shows that the responsivity value does not exhibit a definable change with an increase in excitation intensity for the MoS_2_ nanoflake. The responsivity varies at a small range of 20–30 AW^−1^. The values of the MoS_2_ nanoflake are over three orders of magnitude higher than those (*R* = 0.001 − 0.009 AW^−1^) of the bulk in the intensity range of 50–5000 Wm^−2^.

The MoS_2_ flake device belongs to a two-terminal photoconductor-type photodetector without applied gate bias. Compared to the other 2D material photoconductors, the responsivity values of the MoS_2_ nanoflake are much higher than those of the reduced graphene oxide (*R* = 0.004 AW^−1^) [22], graphene nanoribbon (*R* = 1 AW^−1^) [22], and MoS_2_ multilayer films (*R* = 0.071 − 1.8 AW^−1^) [10–12] and are comparable with the GaS (*R* = 4.2 − 19.2 AW^−1^) [23] and GaSe (*R*~2.8 AW^−1^) [24] nanosheets. On the other hand, three-terminal phototransistors of 2D materials usually exhibit better detector performance due to the field effect. However, if compared to these phototransistors, the responsivity values of the MoS_2_ nanoflake are still higher than those of the graphene (*R* = 0.0005 − 0.0061 AW^−1^) [25, 26] and partial MoS_2_ monolayer (*R* = 0.0075 − 6 AW^−1^) [13, 14] phototransistors but are lower than the optimally reported values of the MoS_2_ monolayer (*R* = 880 AW^−1^) [15], surface-modified MoS_2_ and WSe_2_ (*R* = 5750 − 14,500 AW^−1^) [16], graphene/quantum dots (*R*~10^7^ AW^−1^) [27] and MoS_2_/graphene (*R* = 1.6 × 10^4^ − 5 × 10^8^ AW^−1^) [8, 9, 28] hybrid phototransistors.

It is interesting that the nanostructure has lower photocurrent but exhibits higher photocurrent generation efficiency (i.e., responsivity) compared to the bulk. According to the definition of responsivity, the measured photocurrent is divided by the projected area. Though photocurrent of the bulk is two orders of magnitude higher than that of the nanoflake, the projected area of the bulk crystal (*A* = 1.1 × 2.5 mm^2^) is six orders of magnitude larger than that of the nanoflake (*A* = 1.2 × 2.2 μm^2^). The analysis mathematically explains that the nanoflake device produces less photocurrent but exhibits higher generation efficiency. However, to further understand the physical origins of the superior photodetector performance in the MoS_2_ 2D structures, photoconductive gain was investigated.

Gain (*Γ*) value conceptually means the circulating number of carrier transport through a photoconductor per unit time before recombination. Therefore, gain is defined as the ratio of carrier lifetime (*τ*) to transit time (*τ*_*t*_) between two electrodes and is written as

2 $$ \varGamma =\frac{\tau }{\tau_{\mathrm{t}}} = \kern0.5em \frac{V}{l^2}\kern0.5em \tau \mu, $$

where *μ* is the mobility [21, 29]. Because gain has a linear relationship with responsivity and photocurrent, the gain value can be estimated according to the equation

3 $$ \varGamma =\frac{\mathrm{E}}{e}\frac{R}{\eta }=\frac{E}{e}\frac{i_p}{\eta P}, $$

where *E* is the photon energy, *e* is the elementary charge, and *η* is the quantum efficiency [29].

To simplifying the calculation, the reflection loss was neglected and thus the quantum efficiency can be expressed as *η* = 1 − *e*^*−αt*^, where *α* is the optical absorption coefficient and *t* is the sample thickness. According to the literatures, the *α* value of the MoS_2_ is approximately 3.5 × 10^5^ cm^−1^ for the absorption wavelength near 532 nm (2.33 eV) [30]. The calculated *η* values are 79 and 100 % for the nanoflake (*t* = 45 nm) and the bulk (*t* = 63 μm), respectively.

The calculated gain as a function of intensity is shown in Fig. 3c. The result indicates the gain values (*Γ* = 66 − 103) of the nanoflake is over three orders of magnitude higher than those (*Γ* = 0.0026 − 0.019) of the bulk. In addition, the gain values have been investigated rarely for the photoconductor-type 2D materials compared to other nanomaterial systems, but these values are higher than those of ZnS nanobelts (*Γ*~0.5) [31], ZnSe nanobelts (*Γ*~0.4) [32], ZnO nanospheres (*Γ*~5) [33], and Nb_2_O_5_ nanobelts (*Γ*~6) [34] and are lower than the optimal reported values for the ZnO nanowire (*Γ* ~2 × 10^8^) [35], SnO_2_ nanowire (*Γ*~8 × 10^8^) [36], GaN nanowire (*Γ*~10^8^) [37] photoconductors, graphene/quantum dot (*Γ*~10^8^) [27], and MoS_2_/graphene (*Γ* = 10^7^ − 4 × 10^10^ AW^−1^) [8, 9, 28] hybrid phototransistors.

Photoconductive gain has a physical meaning of the excess carrier collection efficiency in a photodetector. According to Eq. (2), gain value depends on electrode interspace and applied bias. In this study, the ratio of $\frac{V}{l^2}$ of the nanoflake (*l* = 1.2 μm, *V* = 0.1 V) to the bulk (*l* = 1.1 mm, *V* = 1 V) is approximately 84,000:1. The much higher $\frac{V}{l^2}$ value provides a much shorter transport time of carrier $\left({\tau}_{\mathrm{t}}={\left(\frac{V}{l^2}\mu \right)}^{-1}\right)$ and a higher probability of carrier collection, which is the dominant factor for high-gain transport in the MoS_2_ nanoflakes.

**Fig. 4:**
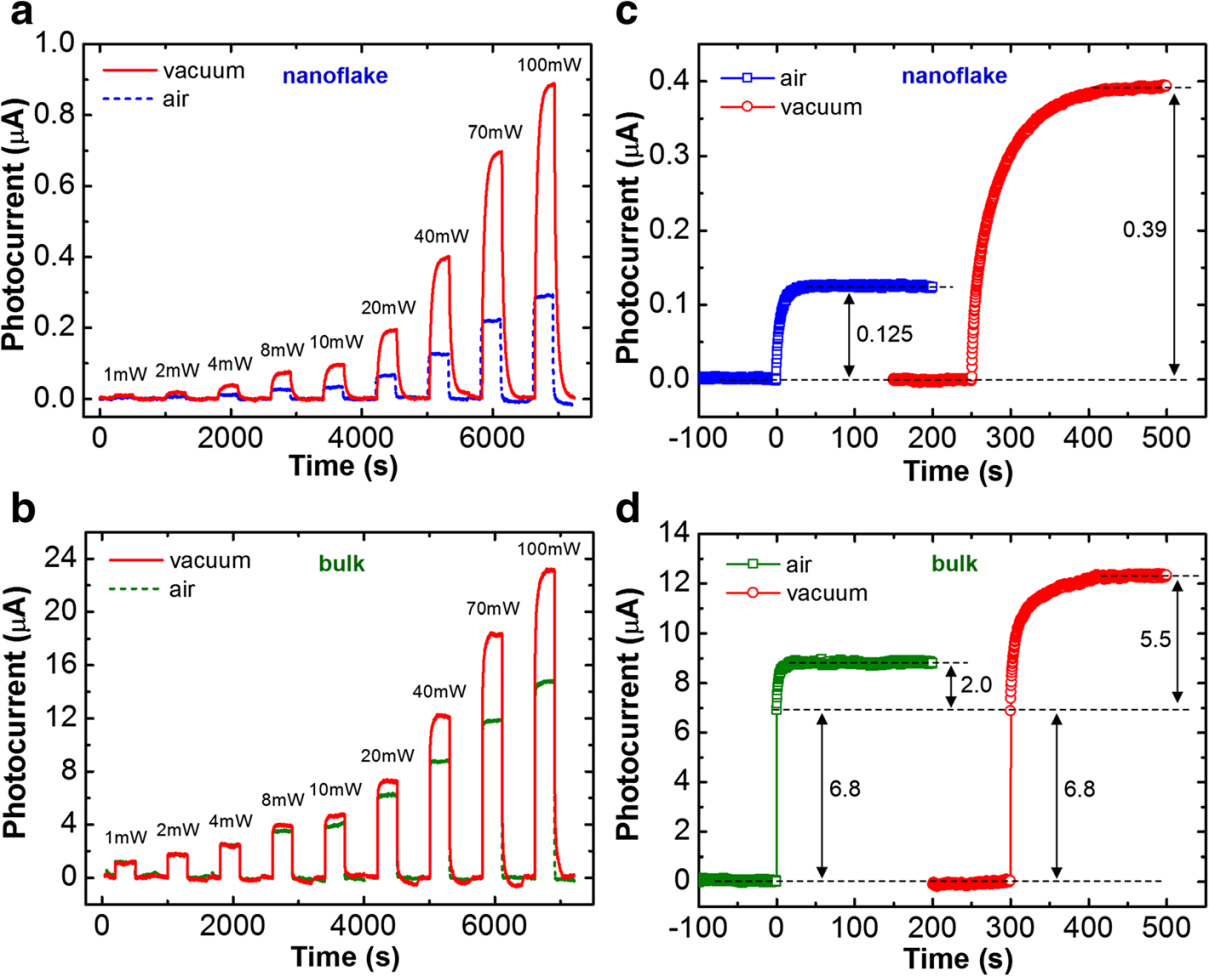
Photocurrent responses to the different light power and the excitation wavelengths of 532 nm measured in air and vacuum ambiences for **a** the MoS_2_ nanoflake (*t* = 45 nm) and **b** the bulk crystal (*t* = 63 μm). Photocurrent rise curves at the excitation power of 40 mW measured in air and vacuum ambiences for **c** the MoS_2_ nanoflake (*t* = 45 nm) and **d** the bulk crystal (*t* = 63 μm)

In addition to the artificial factors of *l* and *V*, gain value could also depend on the *τμ* product which is an intrinsic quantity of a photoconductor. Figure 4a, b illustrates the power-dependent photoresponse curves at the excitation of *λ* = 532 nm measured in atmospheric and vacuum ambiences for the MoS_2_ nanoflake (*t* = 45 nm, *V* = 0.1 V) and the bulk crystal (*t* = 63 μm, *V* = 1 V). The result shows that the photocurrent of the nanoflake can be remarkably enhanced by changing the ambience from air to vacuum. The enhancement of photocurrent in the bulk is relatively less. The ambience-dependent behavior implies a surface-controlled photoconductivity and is similar to the oxygen-sensitized photoconduction (OSPC) mechanism which has been frequently observed in the metal oxide semiconductors [35, 38].

According to the model, carrier lifetime is determined by oxygen (and water molecule) [39, 40] adsorption rate on the material surface. The carrier lifetime can be prolonged in vacuum because of the lower recombination rate induced by the lower oxygen adsorption rate. The prolonged lifetime increases the photocurrent in vacuum. Because the bulk crystal has smaller surface-to-volume ratio, the photocurrent can be generated by both core (ambience-independent) and surface (ambience-sensitive) regions. This statement explains the MoS_2_ bulk with less enhancement of photocurrent in vacuum.

The aforementioned statement can be further supported by the time-resolved photoresponse measurement. Figure 4c, d depicts the photocurrent rise curves at the excitation power of 40 mW measured in air and vacuum ambiences for the nanoflake and bulk. From Fig. 4d, we can notice that the bulk exhibits a two-stage current rise behavior either in air or in vacuum. A slow current rise (with a photocurrent of 2.0 μA and a rise time of 2.7 s) superposes a relatively fast current response (with a photocurrent of 6.8 μA and a rise time less than 0.3 s) in air. The photocurrent and rise time can be both enhanced to 5.5 μA and 16 s, respectively, when changing the ambience to vacuum. However, the photocurrent section of fast response remains constant and is independent on the environment. The result implies that the fast photoresponse originates from the core bulk region, which is different from the slower one. The slow and environment-sensitive properties are consistent with the description of the OSPC mechanism. The ratio of the OSPC photocurrent in air to that in vacuum is approximately 1:3 for the MoS_2_ bulk. The current enhancement ratio is also consistent with that of the nanoflake shown in Fig. 4c. The fast photoresponse was not observed in the MoS_2_ nanoflake, indicating a single surface-dominant photoconduction property.

According to the OSPC mechanism, excess electron lifetime should remain constant because the recombination rate is determined by the oxygen adsorption rate. The constant lifetime is somewhat similar to the hole-trapping mechanism before the trap states are filled [41, 42]. This statement can explain that the responsivity or gain value is independent on the excitation intensity (i.e., *R* or *Γ* ∝ *τ* = *const.*) and the photocurrent is linearly proportional to the intensity (i.e., *i*_p_ ∝ *I*) as observed in Fig. 3. In addition, the bulk exhibits different intensity dependences of photocurrent and responsivity or gain (*i*_p_ ∝ *I*^*β*^, and *R* or *Γ* ∝ *I*^*β*−1^, where *β* = 0.56). The power law dependence is consistent with the bimolecular recombination mechanism in the intrinsic semiconductor in which the *β* value is 0.5 theoretically [41, 43]. The *β* value of the bulk slightly higher than the theoretical one is probably due to the partial contribution of surface PC mechanism.

## Conclusions

Photoconduction performances and mechanisms in the photoconductor-type MoS_2_ nanostructures and bulks were investigated and compared. The responsivity and gain values of the MoS2 nanoflakes are higher than those of the bulk counterparts for several orders of magnitude. An environment-sensitive photoresponse behavior implies the surface-dominant OSPC mechanism in MoS_2_ 2D structures. Further investigations on the intrinsic photoconduction properties such as normalized gain [44] and mobility in the pristine MoS_2_ nanoflakes are still required and will be elaborated elsewhere.

## References

[1] Wang QH, Kalantar-Zadeh K, Kis A, Coleman JN, Strano MS (2012). Electronics and optoelectronics of two-dimensional transition metal dichalcogenides. Nat Nanotech.

[2] Mak KF, Lee C, Hone J, Shan J, Heinz TF (2010). Atomically thin MoS_2_: a new direct-gap semiconductor. Phys Rev Lett.

[3] Splendiani A, Sun L, Zhang YB, Li TS, Kim J, Chim CY, Galli G, Wang F (2010). Emerging photoluminescence in monolayer MoS_2_.. Nano Lett.

[4] Geim AK, Grigorieva IV (2013). Van der Waals heterostructures. Nature.

[5] Withers F, Del Pozo-Zamudio O, Mishchenko A, Rooney AP, Gholinia A, Watanabe K, Taniguchi T, Haigh SJ, Geim AK, Tartakovskii AI, Novoselov KS (2015). Light-emitting diodes by band-structure engineering in van der Waals heterostructures. Nat Mater.

[6] Britnell L, Ribeiro RM, Eckmann A, Jalil R, Belle BD, Mishchenko A, Kim YJ, Gorbachev RV, Georgiou T, Morozov SV, Grigorenko AN, Geim AK, Casiraghi C, Castro Neto AH, Novoselov KS (2013). Strong light-matter interactions in heterostructures of atomically thin films. Science.

[7] Bernardi M, Palummo M, Grossman JC (2013). Extraordinary sunlight absorption and one nanometer thick photovoltaics using two-dimensional monolayer materials. Nano Lett.

[8] Roy K, Padmanabhan M, Goswami S, Sai TP, Ramalingam G, Raghavan S, Ghosh A (2013). Graphene-MoS2 hybrid structures for multifunctional photoresponsive memory devices. Nat Nanotech.

[9] Zhang WJ, Chuu CP, Huang JK, Chen CH, Tsai ML, Chang YH, Liang CT, Chen YZ, Chueh YL, He JH, Chou MY, Li LJ (2014). Ultrahigh-gain photodetectors based on atomically thin graphene-MoS_2_ heterostructures. Sci Rep.

[10] Cho B, Kim AR, Park Y, Yoon J, Lee YJ, Lee S, Yoo TJ, Kang CG, Lee BH, Ko HC, Kim DH, Hahm MG (2015). Bifunctional sensing characteristics of chemical vapor deposition synthesized atomic-layered MoS_2_. ACS Appl Mater Interfaces.

[11] Lu JP, Lu JH, Liu HW, Liu B, Chan KX, Lin JD, Chen W, Loh KP, Sow CH (2014). Improved photoelectrical properties of MoS_2_ films after laser micromachining. ACS Nano.

[12] Ling ZP, Yang R, Chai JW, Wang SJ, Leong WS, Tong Y, Lei D, Zhou Q, Gong X, Chi DZ, Ang KW (2015). Large-scale two-dimensional MoS_2_ photodetectors by magnetron sputtering.. Opt Express.

[13] Yin ZY, Li H, Li H, Jiang L, Shi YM, Sun YH, Lu G, Zhang Q, Chen XD, Zhang H (2012). Single-layer MoS_2_ phototransistors. ACS Nano.

[14] Furchi MM, Polyushkin DK, Pospischil A, Mueller T (2014). Mechanisms of photoconductivity in atomically thin MoS_2_. Nano Lett.

[15] Lopez-Sanchez O, Lembke D, Kayci M, Radenovic A, Kis A (2013). Ultrasensitive photodetectors based on monolayer MoS_2_. Nat Nanotech.

[16] Kang DH, Kim MS, Shim J, Jeon J, Park HY, Jung WS, Yu HY, Pang CH, Lee S, Park JH (2015). High-performance transition metal dichalcogenide photodetectors enhanced by self-assembled monolayer doping. Adv Funct Mater.

[17] Tiong KK, Liao PC, Ho CH, Huang YS (1999). Growth and characterization of rhenium-doped MoS_2_ single crystals. J Cryst Growth.

[18] Li H, Zhang Q, Yap CCR, Tay BK, Edwin THT, Olivier A, Baillargeat D (2012). From bulk to monolayer MoS_2_: Evolution of Raman scattering. Adv Funct Mater.

[19] Chen RS, Tang CC, Shen WC, Huang YS (2015) Ohmic contact fabrication using a focused-ion beam technique and electrical characterization for layer semiconductor nanostructures. J Vis Exp. Article no. e53200, doi:10.3791/53200. 10.3791/53200 PMC4692779 26710105

[20] Chen RS, Tang CC, Shen WC, Huang YS (2014). Thickness-dependent electrical conductivities and ohmic contacts in transition metal dichalcogenides multilayers. Nanotechnology.

[21] Bhattacharya P (1997) Semiconductor optoelectronic devices. Prentice-Hall Inc., New Jersey, Ch. 8, p 346-351

[22] Chitara B, Panchakarla LS, Krupanidhi SB, Rao CNR (2011). Infrared photodetectors based on reduced graphene oxide and graphene nanoribbons. Adv Mater.

[23] Hu PA, Wang LF, Yoon M, Zhang J, Feng W, Wang XN, Wen ZZ, Idrobo JC, Miyamoto Y, Geohegan DB, Xiao K (2013). Highly responsive ultrathin GaS nanosheet photodetectors on rigid and flexible substrates. Nano Lett.

[24] Hu PA, Wen ZZ, Wang LF, Tan PH, Xiao K (2012). Synthesis of few-layer GaSe nanosheets for high performance photodetectors. ACS Nano.

[25] Xia FN, Mueller T, Lin YM, Valdes-Garcia A, Avouris P (2009). Ultrafast graphene photodetector. Nat Nanotech.

[26] Mueller T, Xia FNA, Avouris P (2010). Graphene photodetectors for high-speed optical communications. Nat Photonics.

[27] Konstantatos G, Badioli M, Gaudreau L, Osmond J, Bernechea M, de Arquer FPG, Koppens FHL (2012). Hybrid graphene–quantum dot phototransistors with ultrahigh gain. Nat Nanotechnol.

[28] Chen CY, Qiao H, Lin SH, Luk CM, Liu Y, Xu ZQ, Song JC, Xue YZ, Li DL, Yuan J, Yu WZ, Pan CX, Lau SP, Bao QL (2015). Highly responsive MoS_2_ photodetectors enhanced by graphene quantum dots. Sci Rep.

[29] Razeghi M, Rogalski A (1996). Semiconductor ultraviolet detectors. J Appl Phys.

[30] Beal AR, Hughes HP (1979). Kramers-Kronig analysis of the reflectivity spectra of 2H-MoS_2_, 2H-MoSe_2_ and 2H-MoTe_2_. J Phys C Solid State Phys.

[31] Fang XS, Bando Y, Liao MY, Gautam UK, Zhi CY, Dierre B, Liu BD, Zhai TY, Sekiguchi T, Koide Y, Golberg D, oide D, Golberg (2009). Single-crystalline ZnS nanobelts as ultraviolet-light sensors. Adv Mater.

[32] Fang XS, Xiong SL, Zhai TY, Bando Y, Liao MY, Gautam UK, Koide Y, Zhang X, Qian YT, Golberg D (2009). High-performance blue/ultraviolet-light-sensitive ZnSe-nanobelt photodetectors. Adv Mater.

[33] Chen M, Hu LF, Xu JX, Liao MY, Wu LM, Fang XS (2011). ZnO hollow-sphere nanofilm-based high-performance and low-cost photodetector. Small.

[34] Fang XS, Hu LF, Huo KF, Gao B, Zhao LJ, Liao MY, Chu PK, Bando Y, Golberg D (2011). New ultraviolet photodetector based on individual Nb_2_O_5_ nanobelts. Adv Funct Mater.

[35] Soci C, Zhang A, Xiang B, Dayeh SA, Aplin DPR, Park J, Bao XY, Lo YH, Wang D (2007). ZnO nanowire UV photodetectors with high internal gain. Nano Lett.

[36] Chen RS, Wang WC, Chan CH, Lu ML, Chen YF, Lin HC, Chen KH, Chen LC (2013). Photoconduction efficiencies of metal oxide semiconductor nanowires: The material’s inherent properties. Appl Phys Lett.

[37] Gonzalez-Posada F, Songmuang R, Den Hertog M, Monroy E (2012). Room-temperature photodetection dynamics of single GaN nanowires. Nano Lett.

[38] Chen RS, Chen CA, Tsai HY, Wang WC, Huang YS (2012). Photoconduction properties in single-crystalline titanium dioxide nanorods with ultrahigh normalized gain. J Phys Chem C.

[39] Chakrapani V, Angus JC, Anderson AB, Wolter SD, Stoner BR, Sumanasekera GU (2007). Charge transfer equilibria between diamond and an aqueous oxygen electrochemical redox couple. Science.

[40] Chakrapani V, Pendyala C, Kash K, Anderson AB, Sunkara MK, Angus JC (2008). Electrochemical pinning of the Fermi level: mediation of photoluminescence from gallium nitride and zinc oxide. J Am Chem Soc.

[41] Stevens KS, Kinniburgh M, Beresford R (1995). Photoconductive ultraviolet sensor using Mg-doped GaN on Si(111). Appl Phys Lett.

[42] Binet F, Duboz JY, Rosencher E, Scholz F, Harle V (1996). Mechanisms of recombination in GaN photodetectors. Appl Phys Lett.

[43] Bube RH (1992) Photoelectronic properties of semiconductors. Cambridge University Press, Cambridge. Chap. 2, p 28-30

[44] Prades JD, Jimenez-Diaz R, Hernandez-Ramirez F, Fernandez-Romero L, Andreu T, Cirera A, Romano-Rodriguez A, Cornet A, Morante JR, Barth S, Mathur S (2008). Toward a systematic understanding of photodetectors based on individual metal oxide nanowires. J Phys Chem C.

